# Fiber quality and fertility in male alpacas in the Cusco region of Peru

**DOI:** 10.3389/fvets.2024.1421593

**Published:** 2024-08-21

**Authors:** Joel Pacheco, Fanny Bengtsson, Jakob Killander, Francisco Franco, Nils Lundeheim, Csaba Varga, Renée Båge, Jane M. Morrell

**Affiliations:** ^1^Estación Marangani del Instituto Veterinario de Investigaciones Tropicales y de Altura (IVITA), Facultad de Medicina Veterinaria, Universidad Nacional Mayor de San Marcos, Cusco, Peru; ^2^Clinical Sciences, Swedish University of Agricultural Sciences, Uppsala, Sweden; ^3^Animal Biosciences, Swedish University of Agricultural Sciences, Uppsala, Sweden; ^4^Department of Pathobiology, College of Veterinary Medicine, University of Illinois Urbana-Champaign, Urbana, IL, United States

**Keywords:** fiber diameter, body condition score, age, testosterone, mating behavior

## Abstract

**Introduction:**

High testosterone levels might be associated with coarser fiber in alpacas, affecting fiber quality. In the husbandry systems employed in the Andes, males with higher libido might have an increased frequency of successful matings than males with lower libido. This study aimed to determine some of the factors affecting fiber quality in Peruvian alpacas and to evaluate the association between fiber quality and male mating behavior.

**Methods:**

The study population consisted of 189 adult male alpacas at La Raya, Cusco, Peru, at 4,400 m above sea level, belonging to the National University of San Marcos, Lima, and the National University of San Antonio Abad of Cusco. Fiber samples were collected from male alpacas in September 2015; body condition score (BCS) was evaluated. After washing and drying, the fiber samples were analyzed using an Optical Fiber Diameter Analyser 2000; linear regression analysis between predictor variables (location of alpacas, age, BCS, and fiber color) and fiber quality outcomes was conducted. Fertility data were available only for some males belonging to San Marcos University, consisting of hand-written records from the breeding season January to April 2015; individual fertility quotients were calculated for each male.

**Results:**

Age was associated with fiber quality, young alpacas having the finest fibers (*p* < 0.05). An increased BCS was associated with increased fiber length, fiber diameter and spinning fineness, but decreased comfort factor (*p* < 0.05). White fibers were thinner (*p* = 0.05) than colored fibers, with lower comfort factor and spinning fineness. A significant association between the fertility quotient and fiber curvature was observed (*p* = 0.018).

**Discussion:**

These results suggest that careful selection of breeding individuals and attention to husbandry could result in improved fiber quality among alpaca herds in Peru. However, it would be advisable to increase the number of males studied, using more reliable methods for evaluating male fertility and pregnancy diagnosis than were available for this study.

## 1 Introduction

The indigenous peoples of the Andes are reliant on alpaca herds for their livelihood, both for fiber production and meat (1). Although animals with excellent fiber quality were produced during the Inca period, fiber quality decreased after the Spanish conquest (2), due to loss of knowledge, crossbreeding with llama, and exportation of animals with genes for good fiber quality (3). Many alpaca herders live in harsh environments, below the poverty line; thus, improvements in fiber quality would allow them to escape the poverty trap. The interest in alpacas is increasing around the world (4), both for their fine fiber for use in the textile industry, but also for alpacas as companion and show animals.

Alpaca fiber is renowned for its softness and good textile quality (4). Several characteristics of the fiber are important; for example, comfort factor (CF), the proportion of fibers with diameter <30 μm, gives information on the feel of the fiber when used in a garment. Fiber diameter (FD) is the main criteria when setting the price and determining the use of the fiber (5), and is the major criteria in the selection of breeding animals (6).

According to Peruvian practical standards, fiber fineness is classified in four categories; extra fine ( ≤ 23 μm), fine (23–26.5 μm), semi-fine (26.5–29 μm) and coarse (≥29 μm). Colored fiber are classified in the same way as coarse white fibers (7). Fiber obtained from 1,000 years old El Yaral mummies had mean fiber diameters of 23.6 ± 1.9 and 17.9 ± 1.0 μm, representing fine and extra fine fibers (8). Since these early times, crossbreeding between alpacas and llamas resulted in coarser alpaca fiber in the Andean Plateau region (8). The emphasis on quantity rather than quality by the industry may have contributed to the decrease in fineness of alpaca fibers (7). The unique reproduction characteristics of camelids contribute to the difficulties in improving breeding strategies, while loss of high quality animals to other countries contributes to decreased fiber quality (3).

**Figure 1:**
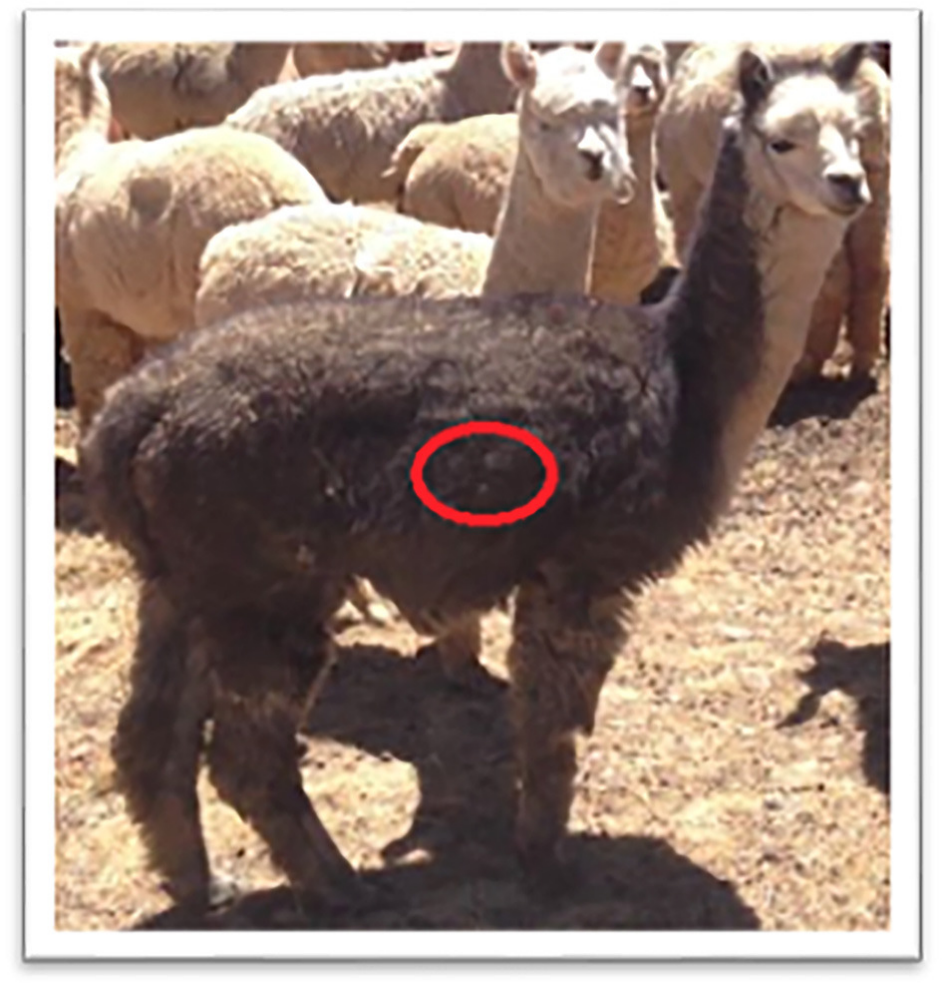
Sampling area on mid-flank region. Photo: Jakob Killander.

The fiber diameter increases with age of the individual (7, 9, 10), although some animals maintain a thin fiber diameter throughout their lives. Gutiérrez et al. (10) found a positive correlation between fiber diameter at birth and the growth of the fiber, although it varies within an animal and between shearing periods. Mayhua et al. (11) reported a slight difference in fiber quality in white alpacas between two shearing periods, December and March, with shearing in December resulting in a better quality fleece. Since fiber diameter varies greatly over the body, samples for analysis should always be taken from the same site. The mid-flank of the animal (Figure 1) is considered to be the most representative area (12).

Fleece color may have an impact on FD. Twenty-two natural colors exist, ranging from white to black (13). Wuliji et al. (14) did not find evidence that colored fleeces have a coarser FD, in contrast to Lupton et al. (15) who found that darker fibers had a higher mean fiber diameter than white and lighter-colored fibers. Similarly, Pinares et al. (16) observed that FD was not affected by different colors in their study on colored fibers.

In sheep, the sex of the animal was observed to affect fiber diameter, with males producing coarser fibers than females because of higher testosterone levels (17). Testosterone concentrations were reported to be higher in dominant breeding stallions than in non-breeding subordinates or in bachelors (18). If a similar effect is seen in alpacas, males with higher testosterone concentrations would produce coarser fibers with a larger diameter.

Alpacas exhibit a distinctive mating behavior. When the male is introduced to a receptive female, he attempts to mount by putting pressure on her pelvis so that she becomes recumbent. If she moves away, the male will chase her for several minutes before she lies down (13). Mating attempts lasting longer than 4 min tend to result in no mating or forced mating (19). The libido and body weight of the male contributes to successful copulation (13). During copulation, the female is in sternal recumbency with her legs beneath her. The male squats to achieve the mating position with his hocks flexed and the metatarsi on the ground, with the front legs stretched forward or bent on either side of the female (20). During copulation, the male makes a special guttural vocalization known as “orgling,” while the female usually remains quiet (19, 20). Copulation lasts, on average, ~20 min, ranging from 5 to 45 min (21, 22), although copulations of different duration did not appear to affect conception (22).

In an uncontrolled mating system, males with high libido will tend to mate with more females than males of lower libido, thus producing more offspring. If testosterone affects fiber diameter in alpacas as reported for rams, males with high testosterone concentration could pass on genes for poor fiber quality to the next generation, resulting in a further decrease in fiber quality in the herd with time. However, to our knowledge, no studies have been published on the relationship between fertility and fiber quality in alpacas. Therefore, the aim of this study was twofold: (i) to determine some of the factors affecting fiber quality in Peruvian alpacas, and (ii) to investigate if there was any association between fiber quality and fertility in alpacas, using the outcome of male mating behavior as a fertility indicator.

## 2 Materials and methods

### 2.1 Animals

The study group consisted of 189 male alpacas of various ages, kept in three groups of 57, 127, and 5 animals, respectively. The groups containing 57 and 5 animals were kept at two locations in the Cusco region, Peru: in the La Raya highlands at 4,200 m above sea level (together with llamas), and on fertile pasture in Fundo, Marangani, at 3,750 m above sea level, respectively. They belonged to the National University of San Marcos. The group of 127 males, also in La Raya, belonged to the National University of San Antonio Abad of Cusco and was also kept together with llamas on richer pasture than for the Lima animals in La Raya.

The alpacas' age in years was calculated from the date of birth to the date of the study. The color was recorded as either white or colored (non-white).

### 2.2 Fiber sampling, washing, and analysis

The fiber is usually removed from the animals in La Raya and Marangani once a year, in September or October, using scissors. Thus, the fiber on the study animals had been cut ~1 year previously.

**Figure 2:**
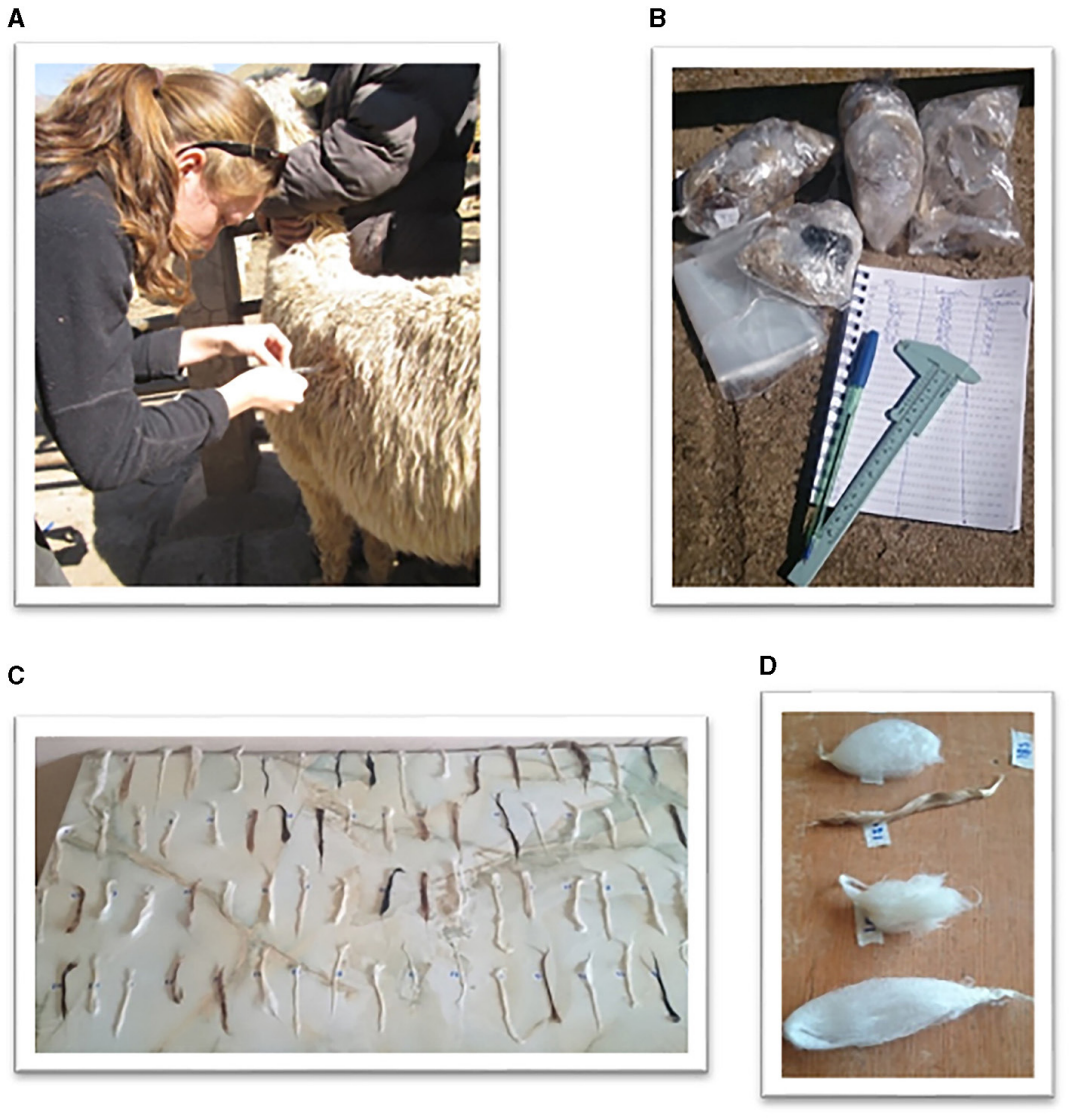
Fiber sampling and processing. **(A)** Fiber length is measured with a caliper (Photo: Joel Pacheco); **(B)** Fiber samples placed in plastic bags for measurement (Photo: Fanny Bengtsson). **(C)** Washed fiber samples drying on a clean table (Photo: Jakob Killander). **(D)** Alpaca fiber samples from Suri (second from top) and Huacaya phenotypes (Photo: Fanny Bengtsson).

An assistant restrained the animals while the fiber was measured at one site mid-flank using calipers (Figure 1) and sampled. Fiber length was straightened by hand and the length measured to the nearest millimeter using calipers (Figure 2A). The fiber was then was cut close to the skin from an area of ~2–4 cm^2^ with scissors. The samples were stored separately in plastic bags for further analysis (Figure 2B).

The fiber samples were washed in a three-steps process. Each sample was washed separately by hand in 30 L of hot (60°C) water containing Opal Ultra washing detergent. After rinsing thoroughly in clean hot water, they were rinsed again in a third sink filled with fresh hot water to ensure that all detergent residues were removed. They were placed horizontally on a clean table in a ventilated room to dry for 3 or 4 days prior to analysis (Figure 2C). The identification number of each samples was written on the table.

When completely dry (Figure 2D), a guillotine was used to cut the samples into 2 mm pieces, which were then spread out on a clean square glass slide and covered with a second glass. Analysis was performed using an Optical Fiber Diameter Analyser (OFDA) laser scanner (OFDA 2000 Benchtop version; BSC Electronics) at the fiber analysis laboratory, University of San Marcos. The instrument was calibrated each year according to the manufacturer's recommendation (23). A video microscope was used to capture and magnify images of the individual fibers for measurement of fiber diameter, curvature value, comfort factor, curvature and spinning fineness.

### 2.3 Body condition score

Body condition score (BCS) was assessed by palpating the muscle mass over the center of the back on each side of the vertebrae, near the last ribs. The scoring system ranged from 1 (very thin) to 5 (obese), including half points. A BCS of 2.5–3.5 is considered to be healthy for all alpacas, although BCS3–3.5 is recommended for pregnant females and young animals (24).

### 2.4 Fertility data

The fertility data consisted of handwritten records from the previous breeding season (January–April 2015), indicating whether a female accepted (A) or rejected (R) the male. Different groups of females were presented to the males on several occasions, ranging from three to six times. The number of occasions was decided in advance by the Peruvian herders and did not depend on the outcome of the attempts (A or R). Results from a maximum of four occasions were included in the present study.

The animals belonging to San Marcos University (Lima) were bred using a controlled breeding system, in which males and females were kept in separate groups during the whole year. During the breeding season, each male mates with 1–8 females, as decided by the herders. One male was let into a smaller pen with the female. If the female spat at the male, “spit-off” or rejection (R) was recorded. If she lay down to allow mating, acceptance (A) was recorded (A). This procedure was repeated at ~14 day-intervals during the breeding season. If a female did not accept the male on one occasion, she was presented with the same male on the next occasion. If she accepted the male on the second attempt, she was considered to have been not pregnant at the previous attempt. Since the females were presented with the same male every time, the records were usable for evaluating the fertility of the males.

Note that although a clinical examination and a breeding soundness examination were performed on the animals as part of the selection of breeding animals, no evaluation of semen quality was possible. Semen collection would be difficult in alpacas under field conditions such as these, where the males are not trained to ejaculate into an artificial vagina and no laboratory facilities are available for sperm evaluation.

### 2.5 Statistical analyses

A fertility quotient was calculated for the males used in the breeding season 2015, using only data from males that mated with more than one female (*n* = 26). Three were of the Suri phenotype and 23 of the Huacaya phenotype; seven were colored and 19 were white. The quotient was calculated from the number of times the females rejected the male divided by the total number of occasions the male was presented to his females. The first occasion on which the female was presented to the male was not included since all females accepted the male on this occasion and were therefore considered to be not pregnant. A maximum of four occasions were included in the study to make the quotients as fair as possible.


$$\text{Quotient}=\frac{\text{Number of rejections}}{\text{Number of occasions }-1}$$

Statistical analysis was performed using the STATA Intercooled software (Version 18, Stata Corporation, College Station, TX). Descriptive statistics was conducted to describe the mean, standard deviation of the mean, and minimum and maximum values of alpacas' age, body condition scores, and various fiber quality parameters.

Individual univariable linear regression models were built to assess the impact of several predictors: herd location (binary variable), age (continuous variable), body condition (continuous variable), color (binary variable) on the following fiber quality outcomes: length, diameter, coefficient of variation, comfort factor, spinning fineness, and fiber curvature.

In addition, univariable linear regression models were built to assess the effect of fiber quality predictors (length, diameter, coefficient of variation, comfort factor, spinning fineness, and fiber curvature) on the outcome variable of fertility quotient.

For each univariable linear regression model a coefficient, its 95% Confidence Interval, and a *p*-value was calculated. The coefficient represents the mean change in the outcome given a one-unit change in the predictor.

Statistically significant associations were implied by a *p*-value of ≤ 0.05. For each statistically significant model outcome, adjusted linear predictions were calculated and illustrated in graphs.

Pearson's correlation coefficients of the pairwise correlations between various paramters of alpaca fiber quality were calculated (length, diameter, coefficient of variation, comfort factor, spinning fineness, and fiber curvature). A significant correlation was represented by a *p*-value of ≤ 0.05.

## 3 Results

The mean, standard deviation of the mean, and minimum and maximum values of age, body condition scores, and various fiber quality parameters are shown in Table 1.

**Table 1:** Descriptive statistics of the age, and body condition score of the alpacas, and various fiber quality parameters.

**Variable**	**Observation**	**Mean**	**SD**	**Min**	**Max**
Age (months)	185	64	33.08816	13	230
Body condition score	188	3.239362	0.682982	1	5
Fiber length (mm)	189	105.381	23.01417	65	206
Mean fiber diameter (μm)	189	26.35577	3.53379	17.96	36.42
Coefficient of variation (%)	189	24.86513	4.3725	16.24	40.48
Comfort factor (%)	189	76.36884	16.69147	22.77	98.71
Spinning fineness (μm)	189	26.61683	3.612406	18.41	37.93
Fiber curvature (°/mm)	189	29.60106	6.780755	9.12	45.45

SD, standard deviation.

**Figure 3:**
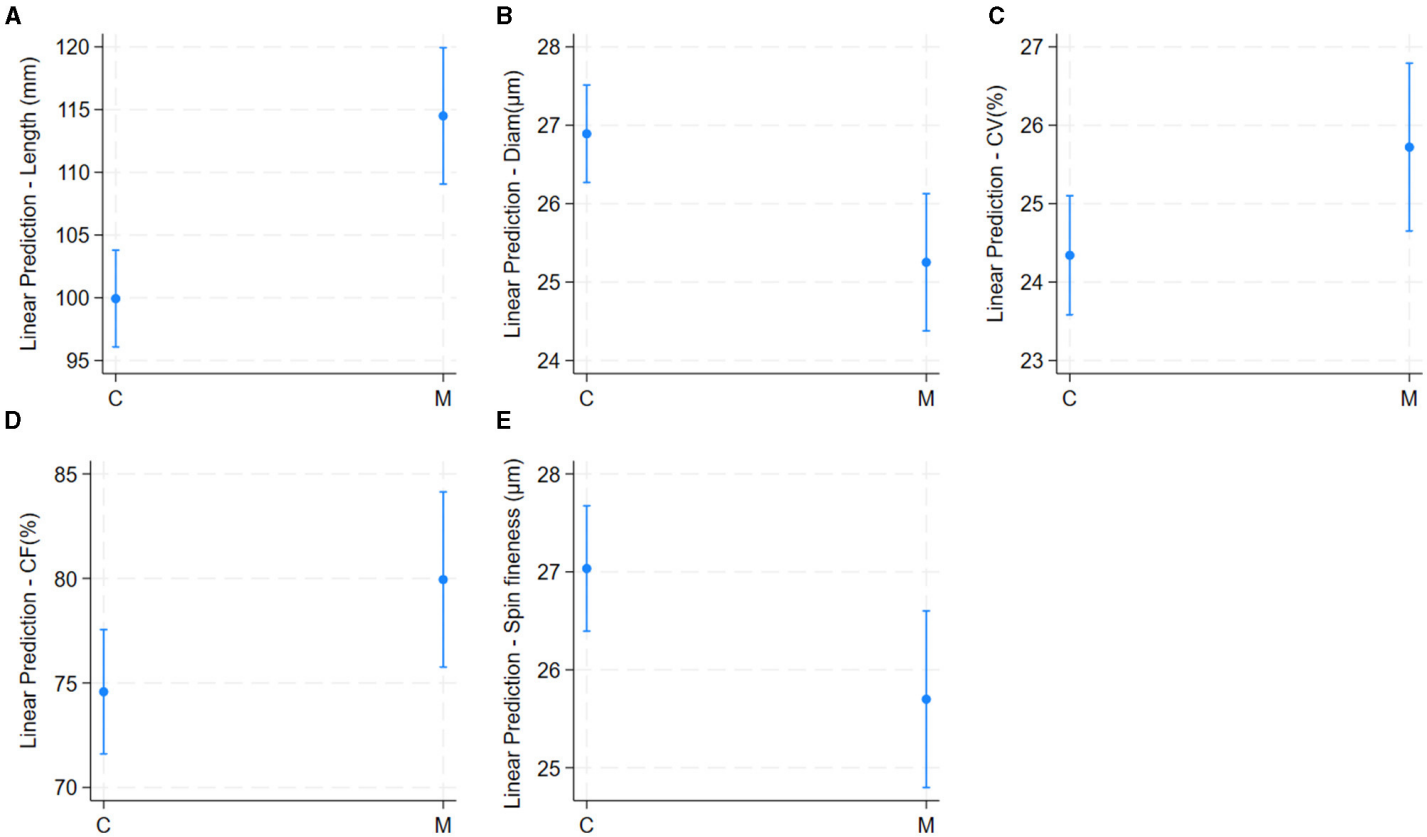
Effect of location of herd (richness of pasture) on alpaca fiber length **(A)**, diameter **(B)**, Comfort Factor **(C)**, spinning fineness **(D)**, and Fiber curvature **(E)**. Adjusted predictions with 95% Confidence Intervals.

The univariable linear regression model results are shown in Table 2. Mean values of fiber quality characteristics in 189 alpaca males are shown in Table 1. Alpacas from the National University of San Marcos had significantly higher fiber lengths, CV, and CF, and lower fiber diameter and spinning fineness than those from the National University of San Antonio Abad (Table 2 and Figure 3).

**Table 2:** Impact of herd location, and alpaca age, body condition and color on fiber quality.

**Predictor variables**	**Fiber quality variables (outcomes)**	**Coefficient**	**95% confidence interval**	***p*-value^a^**
Herd location (1/0)	Length (mm)	14.57	7.90 to 21.23	<0.001
	Diameter (μm)	−1.64	−2.71 to −0.57	0.003
	Coefficient of variation (%)	1.38	0.07 to 2.69	0.039
	Comfort factor (%)	5.37	0.23 to 10.50	0.041
	Spinning fineness (μm)	−1.33	−2.44 to −0.23	0.018
	Fiber curvature (°/mm)	0.87	−1.16 to 2.90	0.400
Age (years) (1–19)	Length (mm)	−2.71	−3.83 to −1.60	<0.001
	Diameter (μm)	0.25	0.06 to 0.43	0.008
	Coefficient of variation (%)	−0.14	−0.37 to 0.08	0.210
	Comfort factor (%)	−0.85	−1.72 to 0.02	0.056
	Spinning fineness (μm)	0.22	0.03 to 0.41	0.022
	Fiber curvature (°/mm)	0.09	−0.26 to 0.43	0.625
Body condition score (1–5)	Length (mm)	9.82	5.12 to 14.52	<0.001
	Diameter (μm)	1.05	0.29 to 1.81	0.007
	Coefficient of variation (%)	0.32	−0.61 to 1.25	0.501
	Comfort factor (%)	−4.28	−7.88 to −0.69	0.020
	Spinning fineness	1.18	0.41 to 1.95	0.003
	Fiber curvature (°/mm)	−4.51	−5.78 to −3.24	<0.001
Color (1 colored/0—white)	Length (mm)	−7.00	−14.32 to 0.32	0.061
	Diameter (μm)	2.82	1.73 to 3.90	<0.001
	Coefficient of variation (%)	1.85	0.47 to 3.23	0.009
	Comfort factor (%)	−12.46	−17.64 to −7.28	<0.001
	Spinning fineness (μm)	3.34	2.26 to 4.42	<0.001
	Fiber curvature (°/mm)	−2.68	−4.79 to −0.56	0.013

Results of univariable (one outcome and one predictor) linear regression models. Herd location was either at the University of San Marco at Marangani, or at the National University of San Antonio Abad of Cusco, at La Raya.

CV, coefficient of variation; CF, comfort factor. Color was either white or colored.

^a^Statistically significant at p ≤ 0.05.

**Figure 4:**
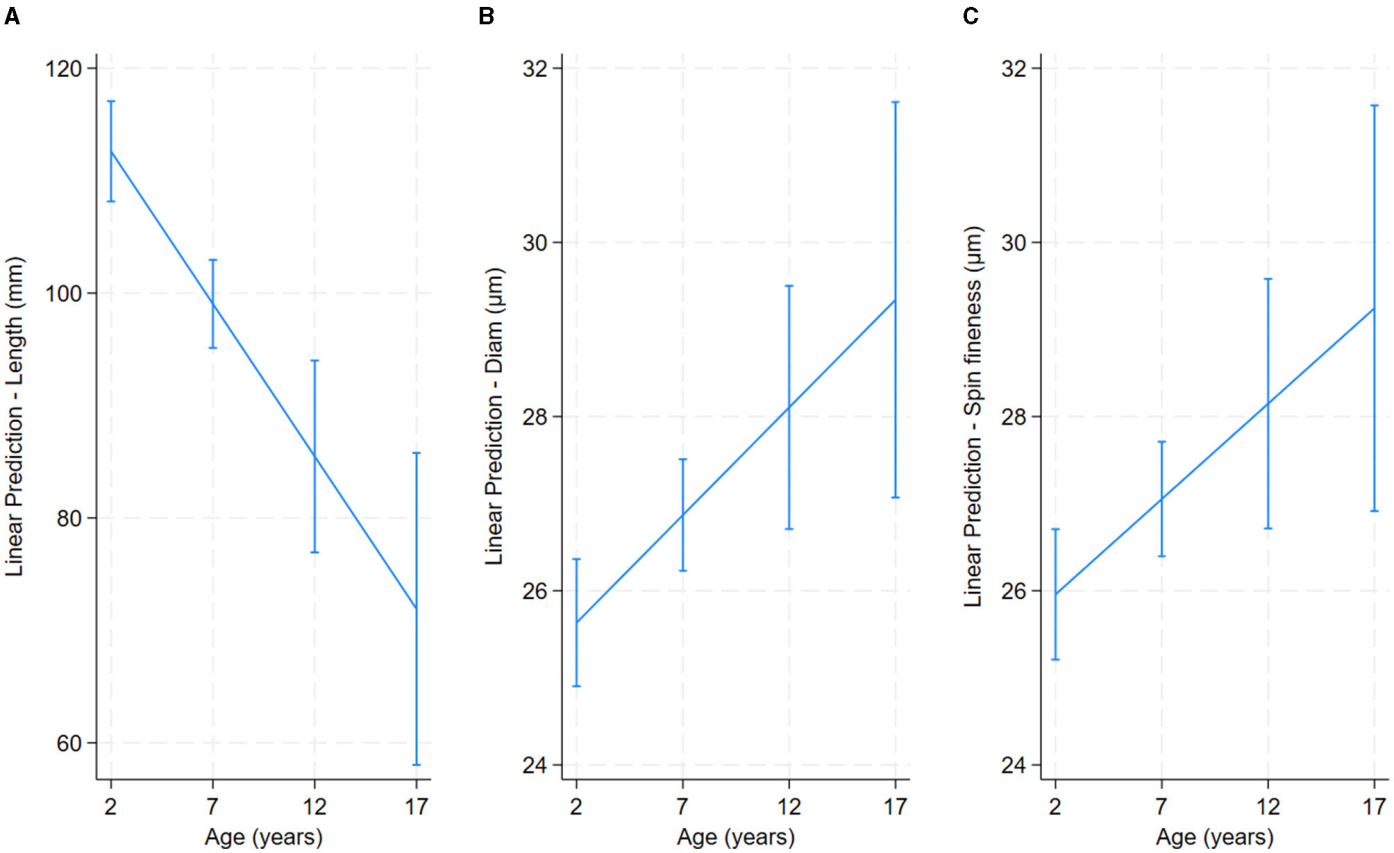
Effect of age on alpaca fiber length **(A)**, diameter **(B)** and spinning fineness **(C)**.

**Figure 5:**
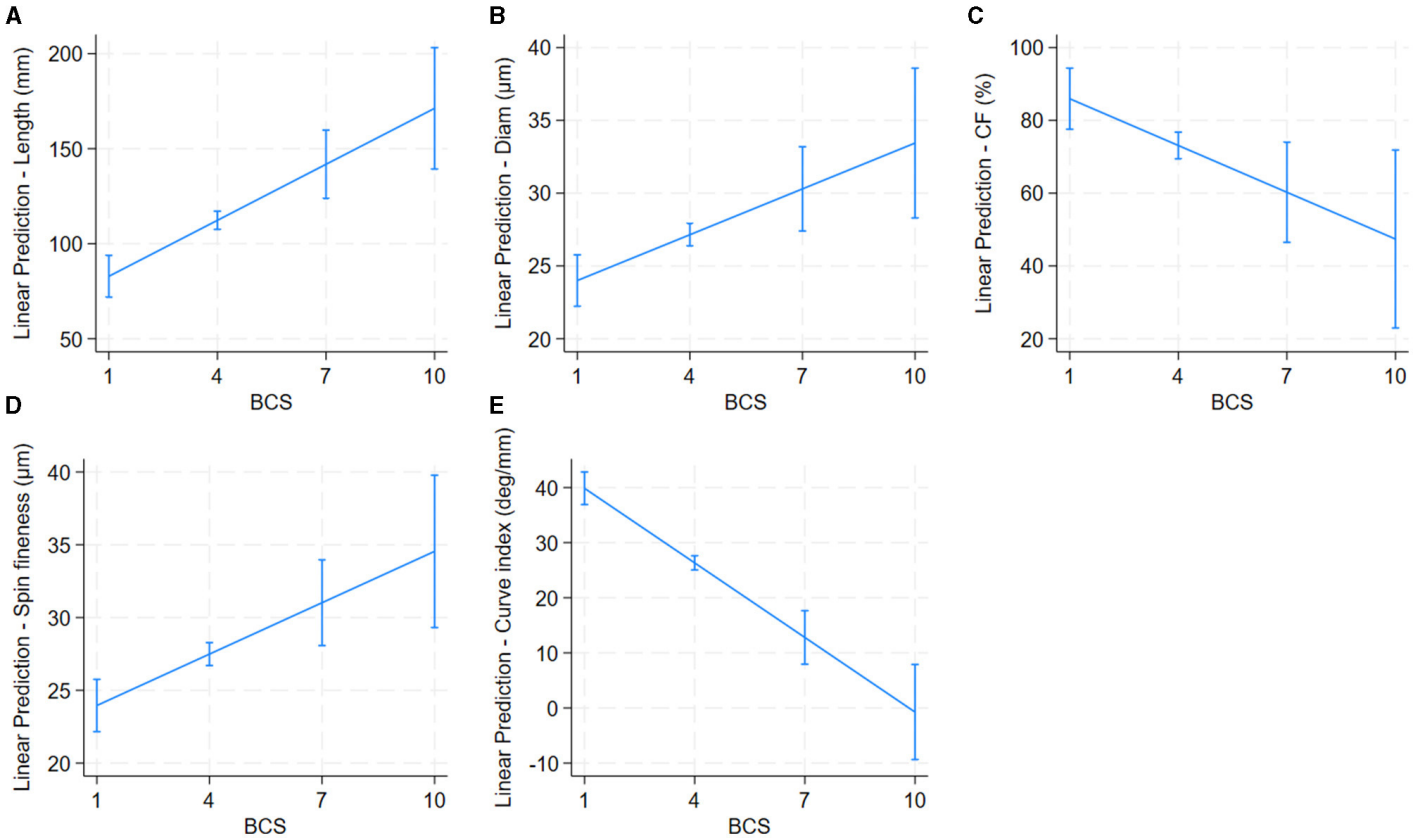
Effect of body condition score (BCS) on alpaca fiber length **(A)**, diameter **(B)**, Comfort Factor **(C)**, spinning fineness **(D)**, and Fiber curvature **(E)**. Adjusted predictions with 95% Confidence Intervals.

**Figure 6:**
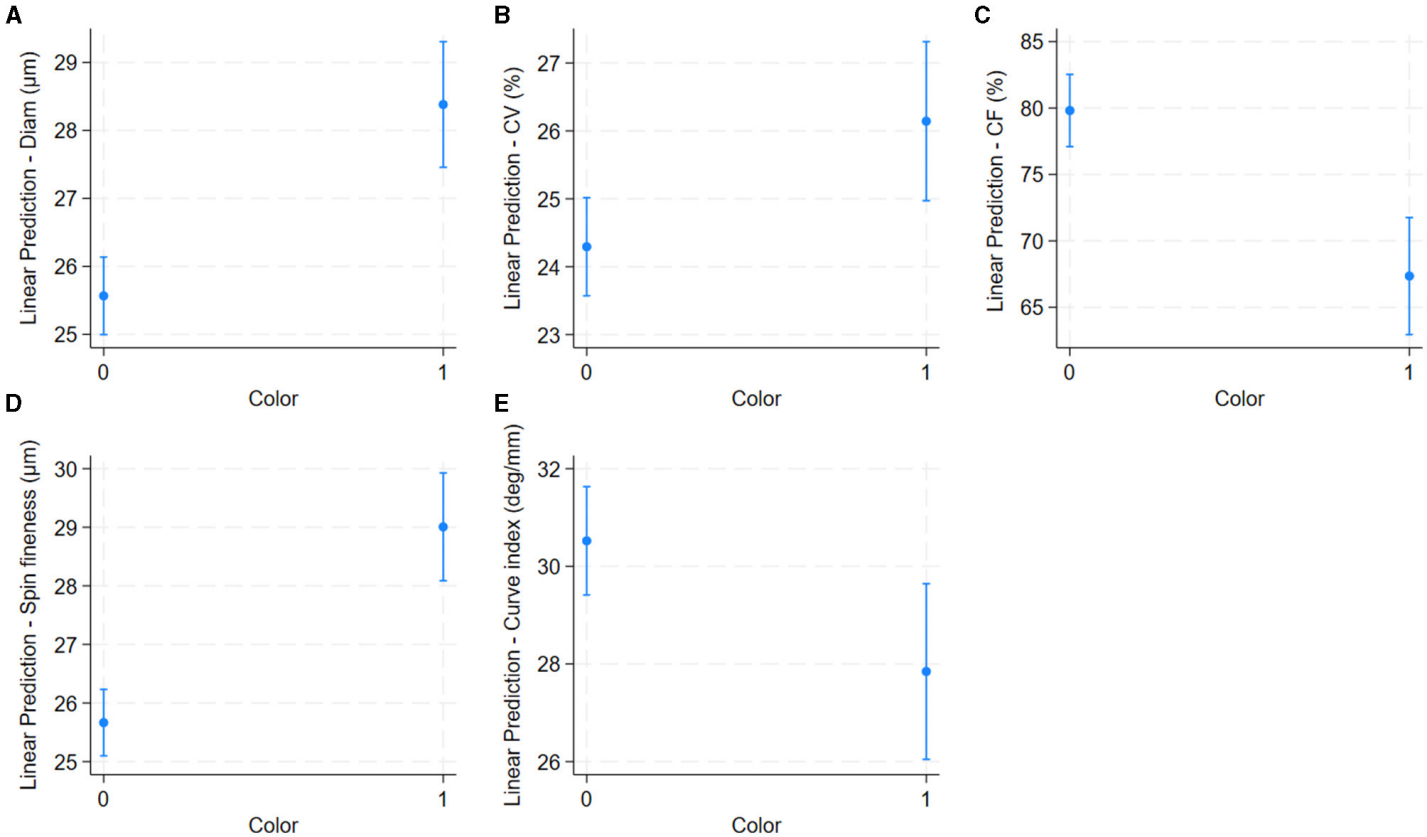
Effect of fiber color (white or non-white) on alpaca fiber length **(A)**, diameter **(B)**, Comfort Factor **(C)**, spinning fineness **(D)**, and Fiber curvature **(E)**. Adjusted predictions with 95% Confidence Intervals.

Older alpacas had significantly higher fiber diameter and spinning fineness, while younger alpacas had longer fiber (Table 2 and Figure 4). Alpacas with higher body condition scores had significantly greater fiber length, diameter, and spinning fineness, while alpacas with lower body condition scores had higher fiber CF and fiber curvature (Table 2 and Figure 5). White alpacas had fibers with significantly lower diameter, CV, and spinning fineness than non-white alpacas, while non-white alpacas had greater fiber CF and fiber curvature (Table 2 and Figure 6).

Pearson's correlation coefficients of the pairwise correlations between various parameters of alpaca fiber quality are presented in Table 3. Fiber diameter was negatively correlated with CV, CS, and spinning fineness, and positively correlated with fiber curvature (Table 3). The fiber–fiber curvature was negatively correlated with fiber length and spinning fineness and positively with CF (Table 3).

**Table 3:** Pearson's correlations among various parameters of alpaca fiber quality.

	**Length**	**Diameter**	**Coefficient of variation**	**Comfort factor**	**Spinning fineness**	**Fiber curvature**
Length	–					
Diameter	• −0.0538 • 0.4667	–				
Coefficient of variation	• −0.0153 • 0.8358	• −0.1434 • 0.0516	–			
Comfort factor	• 0.0331 • 0.6545	• −0.9522 • <0.001	• 0.0488 • 0.5097	–		
Spinning fineness	• −0.0547 • 0.4595	• 0.9508 • <0.001	• 0.1664 • 0.0236	• −0.9266 • <0.001	–	–
Fiber curvature	• −0.3191 • <0.001	• −0.6377 • <0.001	• −0.0065 • 0.9303	• 0.6088 • <0.001	• −0.6374 • <0.001	–

In each cell, the first value is rho, the second is the p-value. Significant at p ≤ 0.05.

**Figure 7:**
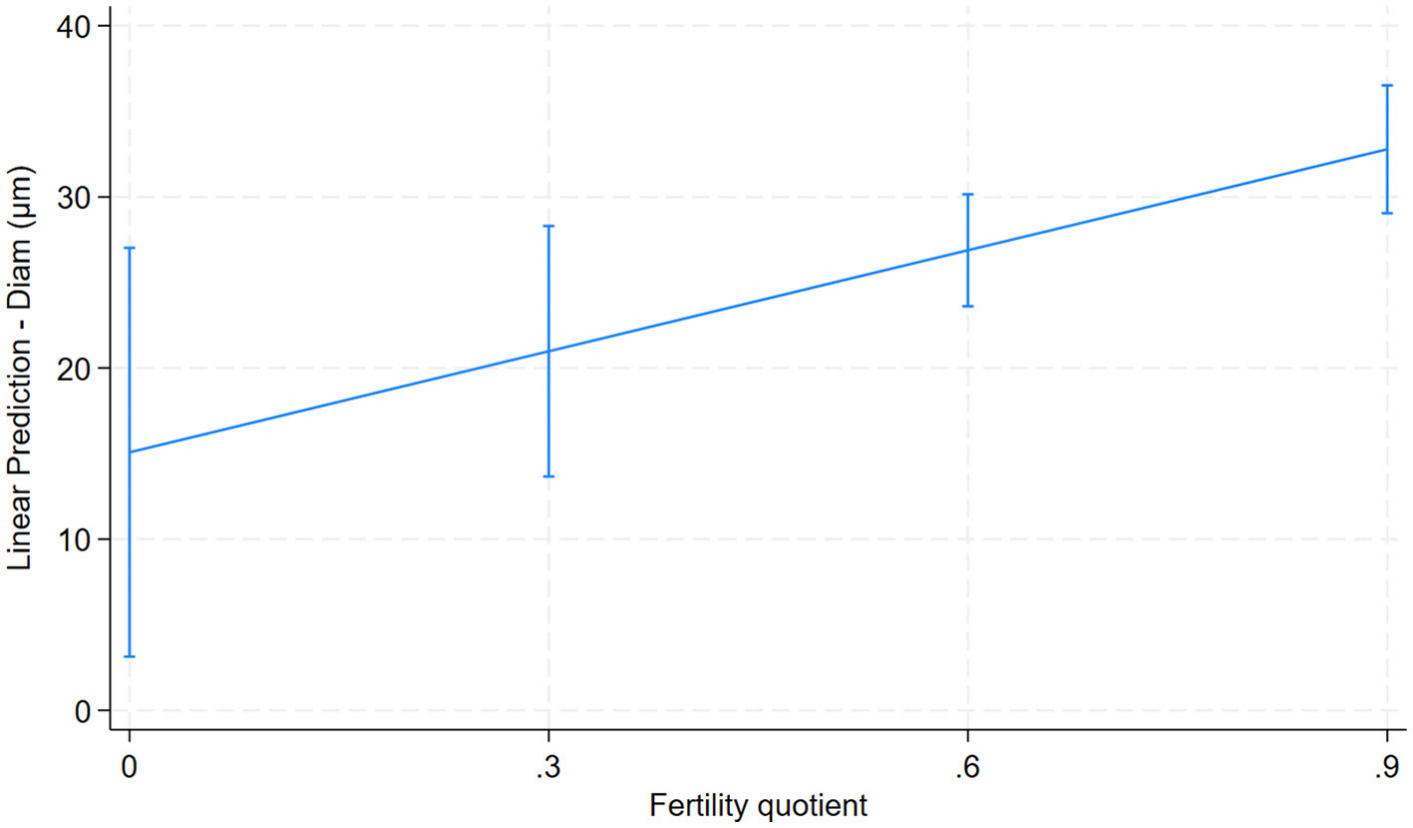
Relationship between fiber diameter and fertility quotient for 26 male alpacas (0.01; 95% CI: 0.002–0.02; *p* = 0.018).

Only one predictor was significantly associated with the fertility quotient. An increase in fiber–fiber curvature (predictor variable) was associated with an increase in their fertility quotient (0.01; 95% CI: 0.002–0.02; *p* = 0.018; Figure 7).

## 4 Discussion

The purpose of this study was to determine some of the factors affecting fiber quality in Peruvian alpacas, and to investigate if there was a relationship between fiber quality and male mating behavior as an indicator of fertility. Various factors impacted fiber quality, particularly age, body condition score and color. The results of this study are in agreement with former studies, where age was associated with fiber diameter (7, 9, 10). In our study, fiber length was significantly affected by age, with the youngest animals having longer fibers and the oldest having shorter fibers. Both Newman and Paterson (25) and Russel and Redden (26) found that a higher level of feeding resulted in increased fiber length, although there was no increase in mean fiber diameter. Similarly, in our study, the animals on richer pasture (the herd from the National University of San Antonio Abad of Cusco) had longer fibers than those of the National University of San Marcos; CV, CF and SF were also affected, the latter negatively. These results correspond to those of Pinares et al. (16) who observed a difference in fiber quality according to location of alpaca herds within the Peruvian Andes. Interestingly, the younger animals in our study did not have a lower BCS than older animals, suggesting that the plane of nutrition was similar for all age groups. In sheep, Masters and Ferguson (27) associated increased stocking rate with decreased live weight gain and FD; they suggested that a strategy for fine fleece production is not necessarily compatible with optimal carcass composition or reproduction, assuming that the increased stocking rate led to less feed intake. It is not known whether nutrition influences fiber quality in the same way in alpacas as in sheep. Certainly, the animals belonging to UNSAAC (with higher FD) grazed on richer pastures and had higher BCS than the animals belonging to UNMSM, which would be expected to result in increased FD. However, they were 22.82 months older on average, which would also result in an increased FD.

Staple length and curvature of the fibers were influenced by BCS, whereas color influenced fiber diameter, comfort factor and spinning fineness. White alpacas consistently showed higher fiber quality than colored animals. The demand for white fibers within the textile industry is higher than for colored fibers because of its ability to be dyed to any color, although there is also a demand for natural colors. Montes et al. (7) considered that pressure to increase the production of white fibers might be a possible reason for an overall decline in fiber quality. There might have been a greater selection for fine fiber within white alpacas in this region, simply because there is a wider range of white animals to choose from White and colored fibers might differ in FD for reasons other than genetics. Wuliji et al. (14) did not find a significant difference in fiber diameter among colored fibers, while Lupton et al. (15) found small but significant differences, with dark fibers being coarser than light fibers. McGregor and Butler (28) reported a 1 μm difference between light and dark fibers, with the light fibers being finer. Radzik-Rant et al. (4) also noted that light fibers were thinner than dark fibers for alpacas in Poland. Cruz et al. (29) observed that although 22 shades of fiber color could be discerned by the human eye, only certain shades should be considered, to avoid observer discrepancies. Apart from white, these are black, brown, dark brown black, gray, light brown and light fawn intensity X, Y, and Z (29). The difficulty in differentiating between colors could have impacted previous studies on color and fiber quality. The division into white and colored in the present study (and some of the previous studies) should have allowed an accurate assessment, since differentiating various shades of color was not required. Originally, it was intended to look at individual colors but there were too few alpacas of each color to allow meaningful statistics on fiber quality to be performed; most colors were represented by ≤ 7 individuals.

Montes et al. (7) and Radzik-Rant and Wiercińska (30) concluded that females had coarser FD than males, while Aylan-Parker et al. (12) observed the opposite. However, in the study by Montes et al. (7), the males were selected breeding animals whereas the females were of different genetic origin. Other authors have found no significant difference in fiber quality between the sexes (14, 15). Lupton et al. (15) reported a difference between sexes in fiber strength, where male alpacas had stronger fibers than females. This was the only fiber characteristic affected by sex in their study, where mean diameter and staple length among other characteristics were measured. Radzik-Rant and Wiercińska (30) considered that light-colored fiber was thinner than dark-colored fiber in Polish alpacas, which was also the case in the Peruvian alpacas in the present study.

Of the correlations between various fiber characteristics, the only fiber characteristics that were not correlated with FD were fiber length and CV (although the latter showed a trend toward significance). These results are in agreement with McGregor (31), who observed that staple length was not altered in association with changes in fiber diameter, and Frank et al. (32), who reported that most fiber characteristics were related to fiber diameter. Our results for CF for white alpaca fibers being related only to fiber diameter differed from those of Pinares et al. (16), who reported that the CF was highly correlated with other characteristics of colored fibers.

Fleece quantity in sheep is related to sex but may be a function of the overall increased body size of rams compared to ewes rather than due to fiber characteristics (33). However, Pinares et al. (16) did not find a signficiant difference in fiber diameter between male and female alpacas.

Literature on the relationship between fiber quality and fertility in alpacas is scarce, but there are some reports of an association in other species. Thus, Safari et al. (34) compiled results from several studies regarding genetic parameters for wool and fertility, among other traits, in sheep. Few estimates of the correlation between reproduction and fiber diameter and staple length were found in the literature, and they were generally low. All correlations between wool fleece weight and fiber diameter and the different reproduction parameters were close to zero. According to Adams and Cronjé (35), maternal factors are more likely to contribute to reproductive disadvantages in fine wool sheep than male reproduction traits, and no relationships between fiber diameter and ram fertility, poor sperm transport or early embryonic mortality were reported. In contrast, Allain and Reniera (36) reported that fleece quality and quantity in small ruminants were due to several traits that were moderately or strongly inherited, suggesting that improvements in both parameters are possible and genetic progress can be made.

No relationships were found between fertility quotients and most aspects of fiber quality, apart from the fiber curvature, in the alpacas in this study. However, fiber curvature increased with increasing fertility quotient. Thus, in our study, the success of the mating attempts was linked to fiber curvature, which was positively related to the comfort factor and negatively related to fiber length, diameter and spinning fineness in the whole study group. Thus, the more “macho” males had shorter fibers, lower fiber diameter, and lower spinning fineness than the less “macho” males. This result is interesting and warrants further investigation. However, several factors made the evaluation of fertility difficult. The animals included were based on “convenience” sampling i.e. breeding was organized according to the needs of the alpaca herders rather than for this study. It was only possible to recover useful breeding data from one site, which dramatically decreased the number of males available. In addition, a different male was used on occasion in the controlled breeding system, if the female was not pregnant and it was assumed that this was due to the male. Therefore, the data from this second group are not included here.

No ultrasonography or other diagnostic aid other than “spit-off” was used to identify pregnant females. Pregnant females may accept the male again under certain circumstances, for example, if he is particularly aggressive or if she is very submissive. Furthermore, not all females that reject the male are pregnant; embryonic loss could have occurred although the regression of corpus luteum had not yet occurred (19). Therefore, using this method alone to verify pregnancies is not sufficiently accurate; it is also difficult to interpret why a female who has rejected the male on one occasion accepts him again on the next attempt. Furthermore, the study described here was performed during September and October, which is not the natural breeding season for alpacas in the Andes; females may refuse to mate at this time of year (37). The number of times that females were presented to the males varied, ranging from three to six times, although results from a maximum of four occasions were included in the study. Some males mated many more females than others (ranging from 1 to 8 females), resulting in irregular data collection for the statistical analysis. It would have been useful to include semen evaluation as well as mating behavior, but this was beyond the scope of the present study. Semen evaluation is complicated in alpacas that are not trained for semen collection (38). Other methods of evaluation, such as testicular measurement, may be seasonally dependent and the relationship with semen characteristics is unknown, making it an imprecise method for predicting sperm production (39). Measuring serum testosterone concentration was not an option as this study was conducted at a field station in the Andes and would have required an intimate knowledge of the diurnal fluctuations in this hormone in alpacas as well as the precise timing of sample collection. However, since hormone concentrations are affected by age and nutrition, which may affect fiber quality, it would be difficult to establish cause and effect. Extending the present study to include a larger number of males with a standardized number of mating attempts would be desirable, and following the fiber quality of their offspring could help determine the heritability of this characteristic in Peruvian alpacas.

## 5 Conclusion

Fiber quality was affected by age, BCS, and color in these Peruvian alpacas, as well as herd location. Older alpacas had greater fiber diameter and spinning fineness than younger alpacas, whereas younger alpacas had longer fiber than older animals. A higher body condition score was associated with longer fibers, greater diameter, and spinning fineness, whereas a lower body condition score was associated with higher fiber CF and fiber curvature. White alpacas had significantly higher fiber diameter, CV, and spinning fineness than colored alpacas, while non-white alpacas had greater fiber CF and fiber curvature than white alpacas. Animals on richer pasture had greater fiber diameter and spinning fineness than those on poorer pasture. An association between fiber curvature and male fertility, as measured by females rejecting further mating attempts by the same male, was found. These results suggest that careful selection of breeding individuals and attention to husbandry could result in improved fiber quality among alpaca herds in Peru. However, it would be advisable to increase the number of males studied, using more reliable methods for evaluating male fertility and pregnancy diagnosis than were available for this study.

## Data availability statement

The original contributions presented in the study are included in the article/supplementary material, further inquiries can be directed to the corresponding author.

## Ethics statement

The animal studies were approved by Estación Marangani del Instituto Veterinario de Investigaciones Tropicales y de Altura (IVITA), Facultad de Medicina Veterinaria, Universidad Nacional Mayor de San Marcos, Cusco, Perú. The studies were conducted in accordance with the local legislation and institutional requirements. Written informed consent was obtained from the owners for the participation of their animals in this study.

## Author contributions

JP: Investigation, Methodology, Resources, Supervision, Writing – review & editing. FB: Investigation, Writing – review & editing. JK: Investigation, Writing – review & editing. FF: Resources, Writing – review & editing. NL: Formal analysis, Writing – review & editing. CV: Formal analysis, Writing – review & editing. RB: Project administration, Supervision, Writing – review & editing. JM: Project administration, Supervision, Writing – original draft, Writing – review & editing.
